# Protein phase separation and its role in tumorigenesis

**DOI:** 10.7554/eLife.60264

**Published:** 2020-11-03

**Authors:** Shan Jiang, Johan Bourghardt Fagman, Changyan Chen, Simon Alberti, Beidong Liu

**Affiliations:** 1 Department of Chemistry and Molecular Biology, University of GothenburgGothenburgSweden; 2 Department of Surgery, Institute of Clinical Sciences, Sahlgrenska Academy, University of GothenburgGothenburgSweden; 3 Department of Oncology at the Department of Clinical Sciences, Sahlgrenska University HospitalGothenburgSweden; 4 Center for Molecular and Cellular Bioengineering, Biotechnology Center, Technische Universität DresdenDresdenGermany; 5 Center for Large-scale cell-based screening, Faculty of Science, University of GothenburgGothenburgSweden; Harvard Medical SchoolUnited States; Harvard Medical SchoolUnited States

**Keywords:** phase separation, cancer, membraneless organelle, biomolecular condensate, *S. cerevisiae*, *E. coli*, Mouse, Human, *C. elegans*

## Abstract

Cancer is a disease characterized by uncontrolled cell proliferation, but the precise pathological mechanisms underlying tumorigenesis often remain to be elucidated. In recent years, condensates formed by phase separation have emerged as a new principle governing the organization and functional regulation of cells. Increasing evidence links cancer-related mutations to aberrantly altered condensate assembly, suggesting that condensates play a key role in tumorigenesis. In this review, we summarize and discuss the latest progress on the formation, regulation, and function of condensates. Special emphasis is given to emerging evidence regarding the link between condensates and the initiation and progression of cancers.

## Introduction

Cancer is a complex disease characterized by loss of control over cell growth, proliferation, and death. In addition to sustaining proliferative signaling and an unlimited replicative potential, many cancers cells acquire the ability to evade growth suppressors, resist cell death, induce angiogenesis, and activate invasion and metastasis (Hanahan and Weinberg, 2011). Moreover, cancer cells interact with surrounding stromal cell and remodel the extracellular matrix to construct specific tumor microenvironments that support tumor growth and progression (Egeblad et al., 2010; Kessenbrock et al., 2010; Lu et al., 2012). Of note, genomic instability raises the possibility to introduce genetic mutations, thus it is often crucial for tumorigenesis (Jeggo et al., 2016). The genetic mutations in cancer-related genes frequently lead to the dysregulation of oncogene activity or they inhibit the activity of a tumor suppressor gene, which then drive the oncogenic process forward. Although significant progress has been made in identifying driver mutations and associated oncogenic processes and pathways, the precise pathological mechanisms underlying tumorigenesis are still largely unclear. Increasing evidence now suggests links between oncogenic processes and the process of condensate assembly by phase separation.

In order to achieve spatiotemporal control over complex biochemical reactions, cells must organize proteins and other macromolecules into subcellular compartments. In addition to the classical membrane-bound organelles, such as the endoplasmic reticulum and Golgi apparatus, cells possess various membraneless compartments, including nucleoli, Cajal bodies, and promyelocytic leukemia protein (PML) bodies in the nucleus (Sawyer et al., 2019) as well as stress granules (SGs) or processing bodies (P bodies) in the cytoplasm (Banani et al., 2017; Jain et al., 2016; Sachdev et al., 2019; Wheeler et al., 2016). Recent studies suggest that the assembly of these membraneless compartments is often driven by a physical process known as phase separation (Banani et al., 2017; Boeynaems et al., 2018).

The term biomolecular condensates is now widely used to refer to membraneless intracellular compartments and assemblies (Banani et al., 2017; Shin and Brangwynne, 2017). In contrast to other types of assemblies, condensates have no fixed stoichiometry, they are able to concentrate molecules, and frequently form through phase separation. Phase separation is a process by which a well-mixed solution of macromolecules such as proteins or nucleic acids spontaneously separates into two phases: a dense and a dilute phase. The dense phase has liquid-like properties and enriches certain macromolecules while others are depleted, allowing the dense phase to function as a compartment (Alberti et al., 2019). After their formation by phase separation, liquid condensates can mature into more solid-like states. They can thus adopt a range of different material properties, from dynamic liquid-like droplets to non-dynamic gels and solid amyloids (Woodruff et al., 2018).

Proper condensate formation is essential for homeostasis as it provides cells with spatiotemporal control over protein function. Under some conditions, such as chronic stress, aging, or disease-associated mutations, abnormal or aberrant condensates can form. These condensates have compositions or properties that deviate from those of condensates formed under physiological conditions. Early research focused on cataracts, showing that phase separation of crystallin proteins in the eye lens causes opacification and thus visual impairment (Siezen and Benedek, 1985; Siezen et al., 1985; Tanaka et al., 1977). Excess accumulation of aberrant crystallin condensates during ageing was proposed to drive the formation of cataracts (Benedek, 1997). Recent studies showed that such aberrant phase transitions are also involved in neurodegenerative diseases, affecting neuronal proteins such as Fused in Sarcoma (FUS) in Amyotrophic Lateral Sclerosis (ALS) (Patel et al., 2015), Tau in Alzheimer's disease (AD) (Wegmann et al., 2018) and huntingtin exon 1 in Huntington's disease (HD) (Peskett et al., 2018). For example, patient mutations in FUS were shown to accelerate a transition of reconstituted FUS droplets from a liquid to a solid-like state (Patel et al., 2015). This hardening of FUS condensates is associated with the formation of supramolecular FUS fibrils that have properties of amyloid-like aggregates. Besides neurodegenerative disorders, current research further suggests emerging roles of aberrant condensates in cancer (Alberti and Dormann, 2019; Spannl et al., 2019).

This review summarizes our current understanding and recent findings regarding the formation, regulation, and function of biomolecular condensates and highlights their emerging roles in the pathogenesis of cancer.

### Molecular features driving protein condensate formation

**Figure 1.**
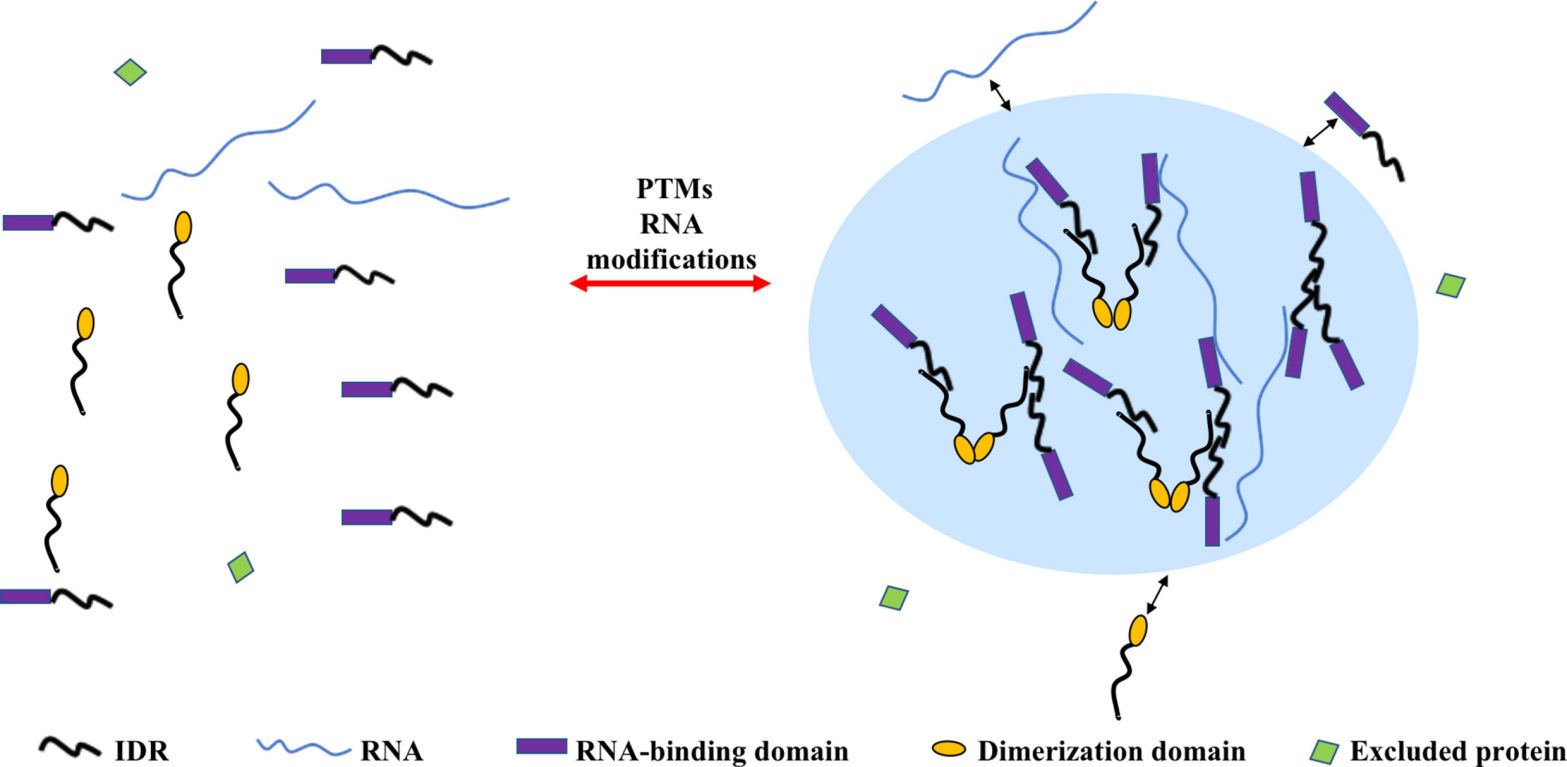
Multivalent interactions drive the assembly of condensates. The diagram depicts different multivalent interactions among proteins or proteins and RNA, which drive the formation of liquid droplets by phase separation. Post-translational modifications (PTMs) (e.g. phosphorylation, acetylation, arginine methylation, and SUMOylation) or RNA modifications (such as m^6^A modification) can increase or decrease the driving forces for phase separation and thus regulate the assembly or disassembly of liquid droplet by phase separation, respectively. Components inside the droplet can exchange dynamically with the dilute phase (indicated by the two-sided black arrows). Some proteins are excluded from the condensate because they do not interact with the molecules in the dense phase (molecule with green domain).

Recent work shows that phase separation requires the establishment of a network of interactions through multivalent protein molecules. These multivalent interactions are promoted by proteins containing multiple-folded modular domains or intrinsically disordered regions (IDRs) (Gomes and Shorter, 2019) or oligomerization domains (Dao et al., 2018; Figure 1). Another class of phase-separating proteins containing polymerizing domains such as the DIX domain, which assemble into filaments that are crosslinked into three-dimensional condensates (Bienz, 2020).

Phase separation by proteins with folded modular domains was first established for two interacting proteins containing multiple copies of the SRC homology 3 (SH3) domains or the SH3 ligand proline-rich motifs (PRM) (Li et al., 2012). The study further demonstrated condensate formation by a three-component system containing the proteins nephrin, NCK, and neural Wiskott-Aldrich syndrome protein (N-WASP), and showed that phase separation by multiple SH3 containing protein NCK and multiple PRM containing protein N-WASP promoted Arp2/3-mediated actin assembly (Li et al., 2012). Multivalent interactions among multi-domain proteins were also shown to underlie the formation of membrane-associated phase-separated signaling clusters by T cell receptor (TCR) components (Ditlev et al., 2019; Huang et al., 2019; Su et al., 2016). The folded modular domains are often connected by intrinsically disordered linker sequences that determine the material properties of the formed condensates (Harmon et al., 2017).

Another class of proteins that has been implicated as drivers of condensate assembly is the class of proteins containing IDRs. Although IDRs lack a defined three-dimensional structure, they often harbor multiple short amino acid motifs that mediate weak interactions. These motifs have been called stickers because of their adhesive properties (Martin et al., 2020; Wang et al., 2018a). The driving forces for sticker-mediated interactions are π-π stacking, cation-π interactions, or charge-charge interactions (Nott et al., 2015; Vernon et al., 2018). The stickers within IDRs are connected by short sequences that are referred to as spacers. Spacers can affect the material properties of condensates. For instance, mutations in spacer residues in FUS changed the material properties of FUS condensates: glycine residues enhanced droplet fluidity, whereas glutamine and serine residues promoted hardening (Wang et al., 2018a).

IDRs that are enriched for only a few amino acids are referred to as low-complexity domains (LCDs) (Boeynaems et al., 2018). A subset of LCDs contains polar, uncharged amino acid residues, such as glutamine (Gln), asparagine (Asn), serine (Ser), or tyrosine (Tyr), and shows a compositional similarity to yeast prion domains (Alberti et al., 2009). These LCDs are referred to as prion-like domains (PLDs) and often found in RNA-binding proteins (RBPs). Proteins with PLDs have initially gained attention because of their ability to assemble into self-templating protein aggregates. These aggregates can have infectious properties, as they can spread between cells, tissues and sometimes individuals (Chakravarty and Jarosz, 2018). More recent studies suggest that PLDs can also act as drivers of condensate assembly. For example, the isolated PLDs of FUS (Burke et al., 2015; Murray et al., 2017), heterogeneous nuclear ribonucleoprotein A1 (hnRNPA1) (Kim et al., 2013) and flowering control locus A (FCA) (Fang et al., 2019) can assemble into condensates at high concentrations in vitro. However, whether PLDs drive phase separation by promoting homotypic PLD-PLD interactions in the cellular context is still unclear. In fact, evidence is emerging that PLDs often have to interact heterotypically with other IDRs during condensate assembly or they modulate the phase behavior of a protein by affecting its overall solubility. For example, phase separation of the FUS proteins was shown to require collective interactions among tyrosine residues in the PLD and arginine residues in the RNA-binding domain (RBD) (Wang et al., 2018a). Moreover, phase separation of the PLD-containing poly(A)-binding protein (Pab1 in yeast) is not mediated by its PLD, but by the RNA recognition motifs (RRMs). Rather, the PLD modulates condensate formation by the Pab1 RRM domains under heat shock condition (Riback et al., 2017). Thus, PLDs may not only be drivers but also modifiers of protein phase behavior (Franzmann and Alberti, 2019).

Finally, multivalent interactions can also arise from oligomerization of folded domains (Bienz, 2020; Dao et al., 2018; Fiedler et al., 2011; Madrzak et al., 2015; Schwarz-Romond et al., 2007). For example, UBQLN2, an adaptor protein required for cellular protein quality control, oligomerizes via the folded STI1-II domain and this promotes UBQLN2 phase separation (Dao et al., 2018).

One important question in the context of this review is whether proteins encoded by cancer-related genes (such as tumor-suppressors or oncogenes) can undergo the above-mentioned multivalent interactions and whether these interactions are perturbed in tumorigenesis. On the one hand, cancer-related mutations could disrupt the assembly of functional condensates by tumor-suppressors, thus contributing to the initiation of cancers. On the other hand, cancer-related mutations could promote the assembly of aberrant condensates by oncoproteins, which might stimulate tumorigenesis.

### Regulation of condensate assembly

Great advances have been made in our understanding of the regulation of condensate assembly. Accumulating evidence suggests that phase separation is often regulated by post-translational modifications (PTMs) (Figure 1). PTMs, including phosphorylation, acetylation, arginine methylation, and SUMOylation, have been implicated in the assembly and disassembly of condensates, as well as the regulation of their material properties (Hofweber and Dormann, 2019). For example, recent studies showed that lysine acetylation regulates phase separation of the microtubule-binding protein Tau (Carlomagno et al., 2017; Ferreon et al., 2018). Moreover, phosphorylation of serine residues or methylation of arginine residues can suppress phase separation of FUS (Hofweber et al., 2018; Monahan et al., 2017; Qamar et al., 2018). These PTMs may play an important role in the formation of pathological FUS aggregates, because many disease-associated mutations in FUS are adjacent to or directly affect amino acids that are modified by PTMs. Importantly, PTMs change not only the driving forces for condensate assembly, but also the selective partitioning of a protein into a condensate. For example, phosphorylation of the RNA polymerase II (Pol II) C-terminal domain prevents the partitioning of Pol II into transcription initiation condensates but it promotes partitioning into splicing condensates (Guo et al., 2019). SUMOylation is another additional PTM that promotes phase separation. For instance, sumoylation of SOP-2 results in an increase in both size and number of condensates in comparison to the unmodified protein (Qu et al., 2020). PTM could change the physicochemical properties of the modified amino acids, which could directly affect multivalent interactions. For instance, phosphorylation attaches a phosphate group to a hydroxyl group of an amino acid side chain, thus introducing a charge that may allow this amino acid to participate in long-range electrostatic interactions.

There now is also increasing evidence for enzymes that specifically regulate the assembly of condensates via PTMs. One example is the dual specificity tyrosine-phosphorylation regulated kinase 3 (DYRK3). DYRK3 localizes to condensates and phosphorylates multiple serine and threonine residues within IDRs (Rai et al., 2018). DYRK3 kinase activity is essential for regulating disassembly of SG during stress recovery (Wippich et al., 2013), and it acts as a dissolvase of many additional membraneless condensates during mitosis (Rai et al., 2018). Many cancer-associated proteins localize to condensates and their localization and activity are regulated by PTMs, suggesting that aberrant condensate regulation by PTMs may be an important pathomechanism underlying cancer.

Another way to regulate the phase behavior of a protein is by regulating the availability of a ligand. For instance, RNA is a ligand of many phase-separating RBPs and it often regulates RBP phase behavior. The effect of RNA on RBP phase separation is concentration-dependent: low amounts of RNA often promote phase separation, whereas high amounts can inhibit it (Maharana et al., 2018). For some RBPs, this effect is independent of RNA sequence, while for others specific RNAs sequences are required (Langdon et al., 2018). Thus, it is conceivable that changes in RNA levels in cancer cells could affect the assembly of condensates. This is particularly true for non-coding RNAs (ncRNA), which are often required for the formation of RNA-protein condensates. One example is ncRNA NEAT1, which is essential for paraspeckle assembly (Yamazaki et al., 2018). More generally, ncRNAs play important regulatory roles in different cancer-related cellular processes and pathways (Anastasiadou et al., 2018). It will be interesting to determine whether altered expression patterns of ncRNAs as they are often observed during tumorigenesis mediate their effects via changes in condensate formation.

Another ligand that has been implicated in condensate regulation is polyADP ribose (PAR). PAR chains are chemically similar to RNA and they promote the condensation of FUS at low concentrations in vitro (Patel et al., 2015). Synthesis of PAR chains at the DNA sites is also required for the recruitment of FUS and the formation of DNA damage condensates in cells (Altmeyer et al., 2015; Patel et al., 2015). These data suggest that the availability of PAR regulates condensation of FUS and potentially many other PAR-binding proteins in cells. Critically, aberrant PAR assembly has been linked to many forms of cancer (Hou et al., 2019).

Studies have also implicated post-transcriptional modifications of RNAs in the regulation of phase separation (Figure 1). N^6^-methyladenosine (m^6^A) is one of the most prevalent types of mRNA modification in cells. Phase separation of the m^6^A-binding proteins YTHDF1, YTHDF2, and YTHDF3 was markedly enhanced by multiple m^6^A modifications on mRNA (Gao et al., 2019; Ries et al., 2019). Moreover, m^6^A modification of mRNA further enhances mRNA partitioning into different condensates. Emerging evidence also suggests that dysregulation of RNA modifications is closely associated with various human cancers (Huang et al., 2020). Notably, the expression of some oncogenic or tumor-suppressive transcripts is regulated by RNA modifications. One possibility emerging from these considerations is that these RNA modifications lead to aberrant condensate assembly and this could result in alterations of oncogene or tumor suppressor gene expression.

### Functions of condensates

Although the functional spectrum of phase separation has not been fully explored, several key functions of condensates have been revealed. For instance, it has been proposed that phase separation of proteins can be used to sense changes in the environment and that the formed condensates then mount adequate adaptive responses. Indeed, the phase behavior of many proteins is very sensitive to small changes in physical-chemical conditions. For example, phase separation of Pab1 has been observed in response to thermal stress and changes in cytosolic pH (Riback et al., 2017). In addition, condensation of Sup35 is induced by an energy depletion-induced acidification of the cytosol (Franzmann et al., 2018). Another example is the RNA-binding protein Pbp1, which senses the cellular redox state and forms condensates under reducing conditions (Kato et al., 2019). In all these cases, the formed condensates play important roles in cellular stress response and adaptation. For example, Pbp1 condensates are able to sequester and inactivate TORC1, thus coupling the metabolic redox state to TOR signaling (Kato et al., 2019).

Condensates have also been shown to accelerate biochemical reaction kinetics by increasing the specific activity of reactants inside condensates. For example, the formation of miRNA-induced silencing complex (miRISC) condensates is associated with increased deadenylation activity (Sheu-Gruttadauria and MacRae, 2018). Some condensates can also sequester proteins or nucleic acids to store them for later use or downregulate enzymatic reactions. For instance, P bodies have been proposed to be reservoirs for translationally repressed mRNAs (Hubstenberger et al., 2017).

Recent work suggests that condensates can also be used to regulate gene expression. For example, super-enhancers, which are clusters of several hundred enhancers, are bound cooperatively by transcription factors to drive robust transcription of genes for defining cell identity (Hnisz et al., 2013). Several lines of evidence suggest that transcriptional coactivators and the mediator complex form condensates at super-enhancers, which may help to compartmentalize and concentrate the transcription apparatus (Boija et al., 2018; Cho et al., 2018; Sabari et al., 2018). Notably, Michnick and colleagues demonstrated that condensation by endocytic proteins can deform the plasma membrane and drive membrane invagination (Michnick et al., 2019). Very recent evidence also suggests that phase separation can buffer protein concentration noise (Klosin et al., 2020). Moreover, condensate formation has been linked to cargo sorting. In chloroplasts, the interaction among STT complex sorting factors and Tat substrate protein induces the formation of condensates, which is critical for Tat protein transport across the stroma to thylakoid membranes (Ouyang et al., 2020).

Lastly, condensates may work as compartments for protein quality control. Under stress conditions, some misfolded proteins accumulate in the granular component (GC) phase of the nucleolus, which prevents irreversible aggregation of misfolded proteins, facilitating refolding during recovery from stress (Frottin et al., 2019; Mediani et al., 2019). Phase separation has also been shown to be critical for the formation of proteasome-containing foci and the assembly of the autophagosome (Fujioka et al., 2020; Yasuda et al., 2020).

In summary, phase separation appears to touch on almost any fundamental process in cells. However, whether and how condensates affect the onset, progression, metastasis and drug resistance of different cancers is still largely unclear. Given the importance of condensates for normal cellular physiology, it seems reasonable to assume that aberrant condensate assembly is a frequent occurrence in tumorigenesis.

### Condensate assembly by cancer-related proteins

In recent years, some oncogenic processes have been linked to condensates formed by cancer-related proteins (Table 1). These cancer-related proteins are involved in the degradation of oncogenic substrates, maintenance of genomic stability, transcriptional regulation and oncogenic signaling pathways, protein quality control and degradation.

**Table 1.** Cancer-related proteins involved in formation and regulation of condensates.

Biomolecular condensates	Cancer-related protein or RNA molecular	Verification of phase behavior	References
SPOP/DAXX bodies	SPOP	Yes	Bouchard et al., 2018
DNA repair condensates	53BP1	Yes	Kilic et al., 2019
DNA damage condensates	PARP-1	Yes	Altmeyer et al., 2015; Patel et al., 2015
DNA damage condensates	FUS	Yes	Altmeyer et al., 2015; Patel et al., 2015
DNA damage condensates	EWS	Yes	Altmeyer et al., 2015; Patel et al., 2015
DNA damage condensates	TAF15	Yes	Altmeyer et al., 2015; Patel et al., 2015
Transcriptional condensates	EWS-FLI1	Predicted	Boulay et al., 2017
Transcriptional condensates	YAP	Yes	Cai et al., 2019
Transcriptional condensates	TAZ	Yes	Lu et al., 2020
PRC1 condensates	CBX2	Yes	Plys et al., 2019; Tatavosian et al., 2019
Transcriptional condensates	β-catenin	Yes	Zamudio et al., 2019
p62 bodies	p62	Yes	Cloer et al., 2018; Sun et al., 2018; Zaffagnini et al., 2018
Stress granules	KRAS	Unconfirmed	Grabocka and Bar-Sagi, 2016
Stress granules	DDX3X	Yes (only in vitro)	Hondele et al., 2019; Valentin-Vega et al., 2016
Stress granules	YB-1	Unconfirmed	Somasekharan et al., 2015
PML NBs	PML/RARA	Unconfirmed	de Thé et al., 2017; Dos Santos et al., 2013
Paraspeckles	NEAT1_2	Yes	Yamazaki et al., 2018

### Speckle-type POZ protein (SPOP) condensates in degradation of oncogenic substrates

**Figure 2.**
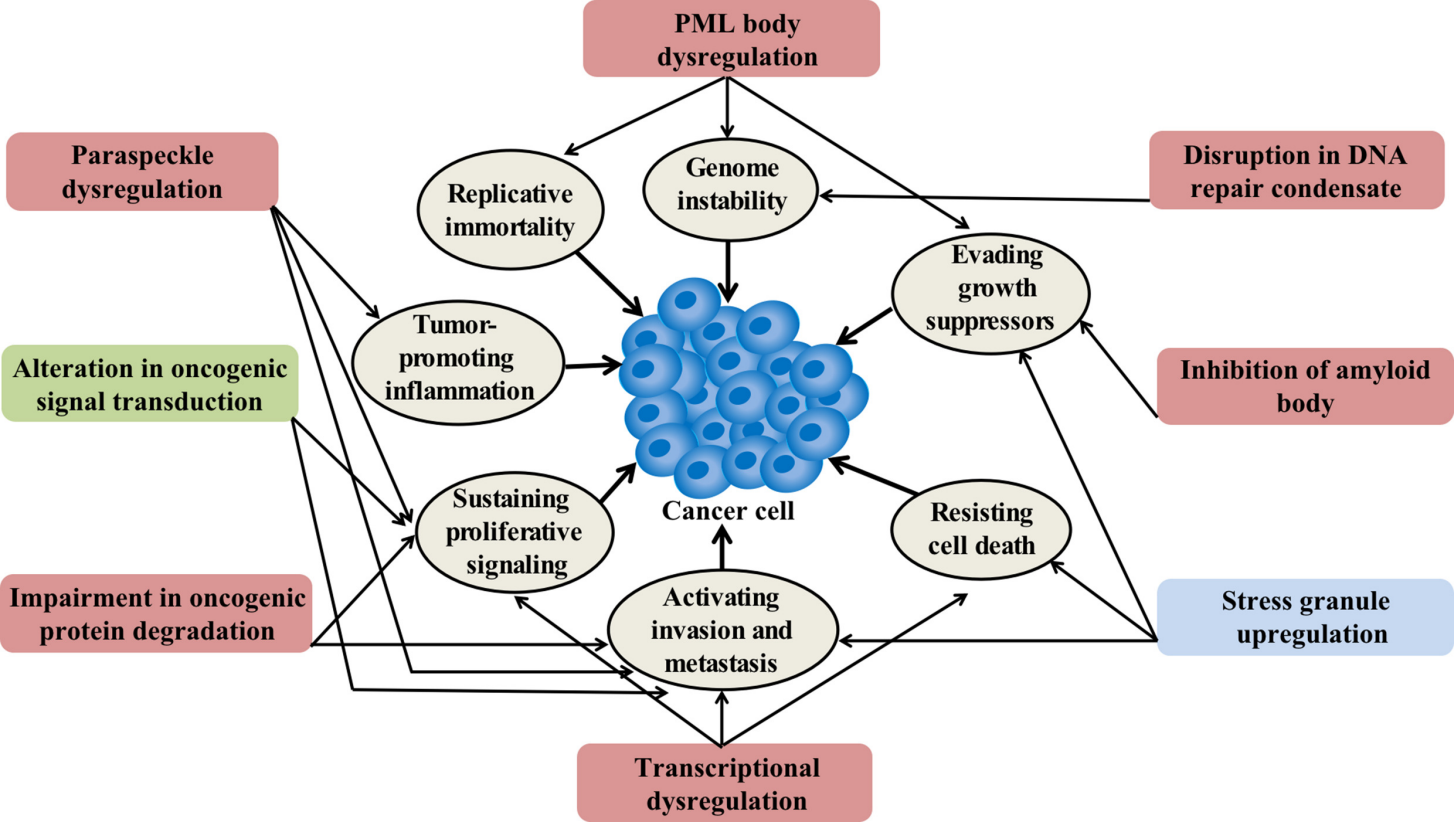
Condensation-related cellular process involved in cancer. Cancer-related proteins may regulate condensate assembly and cancer-associated mutations may alter condensate assembly, thus driving aberrant cellular processes that promote tumorigenesis and cancer progression. Additionally, condensate dysfunction could also play important roles in the development of cancer. Potential links between aberrant condensates and hallmarks of cancer are indicated by arrows. The color of the box indicates the localization of condensates. Red means localize in the nuclear, blue means localize in the cytoplasm, and green means localize in both locations.

The tumor suppressor SPOP functions as a substrate adaptor of the cullin3 (CUL3)-RING ubiquitin ligase complex, which is frequently mutated in prostate cancer (Barbieri et al., 2012; Le Gallo et al., 2012). Tumor-associated missense mutations in the substrate recognition domain of SPOP disrupt substrate binding and ubiquitination, leading to the accumulation of oncogenic substrates, such as steroid receptor coactivator (SRC3), c-MYC (Geng et al., 2017) and death-domain-associated protein (DAXX) (Kwon et al., 2006). Bouchard et al. found that multivalent interactions between SPOP oligomers and motifs in oncogenic substrate proteins drive phase separation in vitro. The same multivalent interactions are required for SPOP co-localization with oncogenic substrates in nuclear condensates. Importantly, substrate proteins such as DAXX appear to be ubiquitylated inside the condensates in a CUL3-dependent manner. Consequently, cancer-associated SPOP mutations disrupt SPOP interaction with the substrates, causing a failure to form condensates, ubiquitylate the substrates and promote their degradation (Bouchard et al., 2018; Table 1 and Figure 2). These findings reveal a direct link between the aberrant phase behavior of a tumor suppressor protein and its downstream effects on oncogenic proteins.

### Condensates in the maintenance of genomic stability

**Figure 3.**
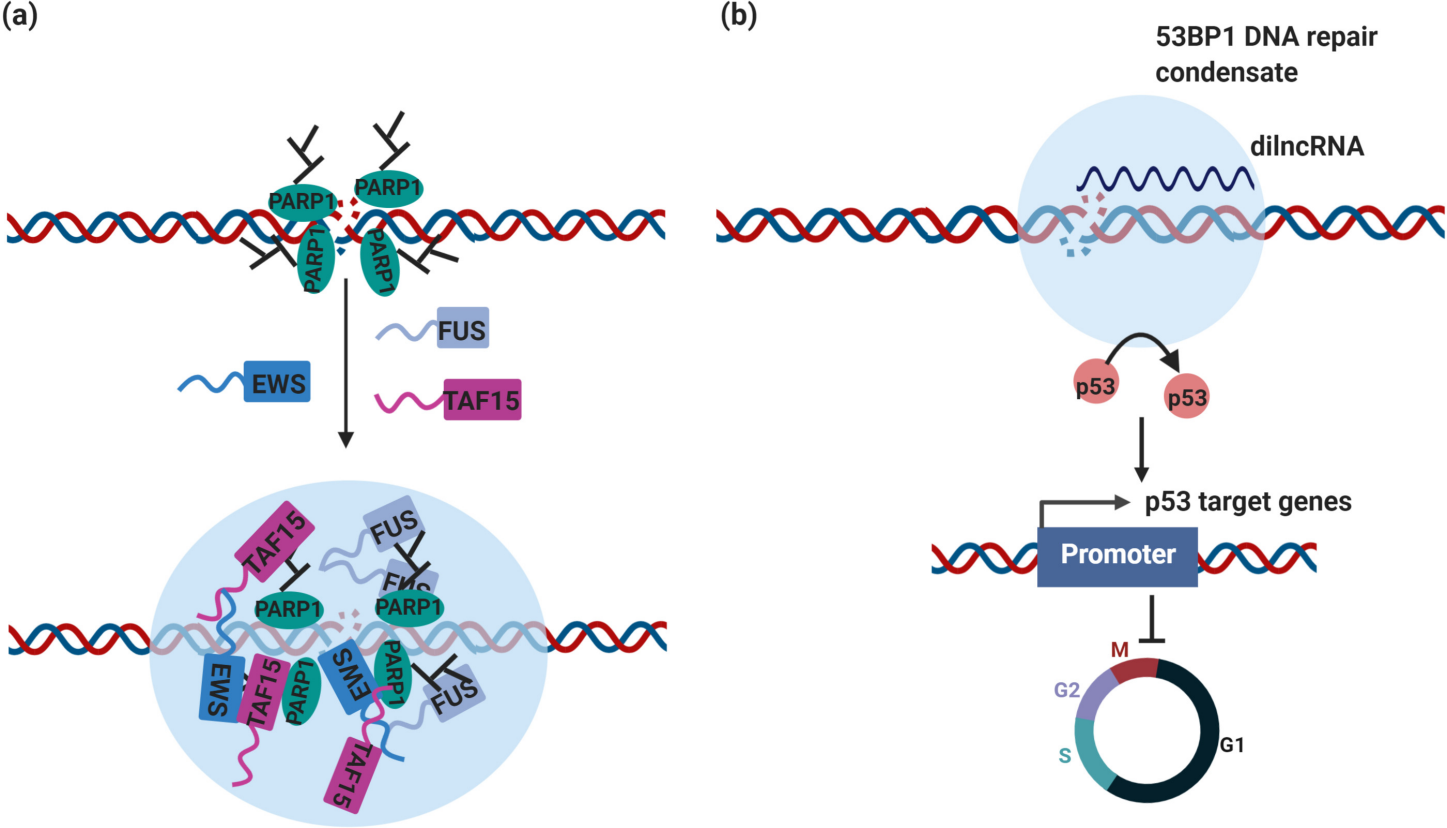
DNA repair condensates in the maintenance of genomic stability. (**a**) DNA damage induces PARylation by PARP around the DSB area, which creates a platform for protein assembly. The recruitment of RBPs containing prion-like domain, such as FUS, EWS, TAF15, drives the assembly of DNA damage condensates at DNA double strand break sites. (**b**) Synthesis of dilnc RNA promotes the assembly of 53BP1 repair condensates at DNA double strand breaks. These condensates recruit p53, which stabilizes p53 and promotes activation of p53 target genes and cell cycle arrest. It is possible and has not been tested yet that these condensates are different stages of DNA damage condensates, condensates in (**a**) are the early stage of DNA damage condensates, condensates in (**b**) are the late stage of DNA damage condensates.

Genomic instability usually arises from disruption of DNA repair and DNA damage response (DDR). Recent studies indicate that condensate assembly is linked to the maintenance of genomic stability. For instance, poly (ADP-ribose) polymerase 1 (PARP1) is one abundantly expressed member of the poly (ADP-ribose) polymerase (PARP) family. PARP1 synthesizes long PAR chains at DNA damage sites and plays a key role in DDR and PARP-1 has been ascribed diverse pro- or anti-tumorigenic roles (Weaver and Yang, 2013). The formation of PAR chains was shown to initiate the formation of DNA damage condensates via recruitment and assembly of FET proteins (FUS, EWS, and TAF15) (Altmeyer et al., 2015; Patel et al., 2015; Table 1; Figure 3).

Additional studies have provided evidence for the involvement of condensates in maintaining genome stability. As one of the main mediators of the DNA damage response (DDR), p53-binding protein 1 (53BP1) acts as a recruitment platform for other DDR proteins (Mirza-Aghazadeh-Attari et al., 2019). 53BP1 protein was first discovered as a binding partner of the tumor suppressor p53 (Iwabuchi et al., 1994; Vousden and Prives, 2009) and plays a direct role in p53 target gene expression, driving a cell toward an anti- or pro-tumorigenic cell fate (Cuella-Martin et al., 2016). Loss of 53BP1 has been associated with poor survival, inhibition of apoptosis, and cancer cell proliferation in colorectal cancer (Bi et al., 2015).

Recently, 53BP1 was reported to drive the formation of a DNA damage repair compartment through phase separation in response to DNA damage, which was promoted by damage-induced long non-coding RNAs (dilncRNA) synthesized at DNA double-strand breaks (DSBs) (Kilic et al., 2019; Pessina et al., 2019; Table 1 and Figure 3). Blocking dilncRNA transcription or inhibiting DNA repair condensate assembly through the chemical 1,6-hexanediol both led to a reduction in the efficiency of DNA repair, suggesting that 53BP1 repair compartment assembly is required for DSB repair (Pessina et al., 2019). Importantly, these DNA damage repair condensates recruit downstream effectors, such as p53 and the p53 co-activator USP28, which stabilizes p53 upon DNA damage. As a consequence, disruption of DNA damage repair compartments led to impaired p53 induction and diminished p53 target gene expression as well as cell cycle arrest (Kilic et al., 2019; Figure 2). Therefore, aberrant DNA damage repair condensate assembly because of alterations in 53BP1 expression is likely to affect the central tumor suppressor protein p53 and thus the expression of many cancer-linked genes.

Intriguingly, 53BP1 is excluded from above-mentioned DNA damage condensates (Altmeyer et al., 2015). Given that 53BP1 is recruited to DNA damage sites later (Aleksandrov et al., 2018), this suggests that cells can assemble DNA damage condensates with different compositions and presumably also functions (Altmeyer et al., 2015). These studies suggest that condensate assembly is intimately linked to the maintenance of genome stability. Indeed, cancer-associated translocations in FET proteins appear to impair the cellular ability to interact with PAR chains, which may affect the assembly of condensates at DNA damage sites and thus undermine genome integrity (Altmeyer et al., 2015).

### Transcriptional condensates in regulation of oncogenic transcriptional programs

Transcriptional dysregulation is a key feature of cancer (Figure 2). Recent studies have implicated condensates in the regulation of oncogenic transcription programs.

EWS-FLI1 is an oncogenic transcription factor that plays key roles in Ewing’s sarcoma tumorigenesis (Delattre et al., 1992). This oncogenic factor is generated by a chromosomal translocation, in which a large portion of the prion-like domain (PLD) of EWSR1 is fused to the transcription factor FLI1 in Ewing’s sarcoma (Tan and Manley, 2009; Toretsky and Wright, 2014). Rivera, Kadoch, and colleagues demonstrated that the translocated PLD was essential for recruiting the BRG1/BRM-associated factor (BAF) chromatin-remodeling complex to tumor-specific enhancers, activating an aberrant transcriptional cascades that is underlying Ewing sarcoma progression (Boulay et al., 2017; Table 1). Moreover, EWS-FLI1 forms numerous nuclear foci, whereas FLI1 exhibits a more diffuse pattern (Boulay et al., 2017). The hypothesis was put forward that this transcriptional program is driven by aberrant condensate formation of EWS-FLI1. In addition, the PLDs of the related proteins FUS and TAF15 are often fused to transcription factors through chromosomal translocations in liposarcoma and acute leukemia, respectively (Tan and Manley, 2009). It is possible that translocated FUS and TAF15 PLDs promote the assembly of additional aberrant condensates at enhancers and promoters, thus driving abnormal tumorigenic transcriptional programs.

Evidence is now also emerging that master transcription factors and the mediator coactivator use their disordered regions to form condensates at super-enhancers, which recruit Pol II to activate transcription sites (Boija et al., 2018; Cho et al., 2018; Sabari et al., 2018). In some cancer cells, genomic alterations promote the formation of super-enhancers at oncogenes, which promote oncogenic transcriptional programs (Sengupta and George, 2017). Thus, aberrant condensation at super-enhancers might be a general mechanism that cancer cells use to sustain high oncogene expression levels.

One example of a transcription regulator implicated in the assembly of a transcriptional condensates and super-enhancer formation is the transcriptional co-activator Yes-associated protein (YAP) and its paralog transcriptional coactivator with PDZ-binding motif (TAZ). YAP and TAZ have been linked to tissue growth, stem cell activity, and tumorigenesis (Moya and Halder, 2019). They bind to a subset of highly active enhancers and super-enhancers to drive the transcription of cell proliferation genes (Galli et al., 2015). Consequently, YAP and TAZ are pervasively activated in cancers (Zanconato et al., 2016; Zanconato et al., 2019). However, YAP and TAZ not only promote to tumorigenesis but also have tumor-suppressive functions. For example, activation of YAP in tumor-surrounding cells can suppress liver cancer in mice (Moya et al., 2019).

**Figure 4.**
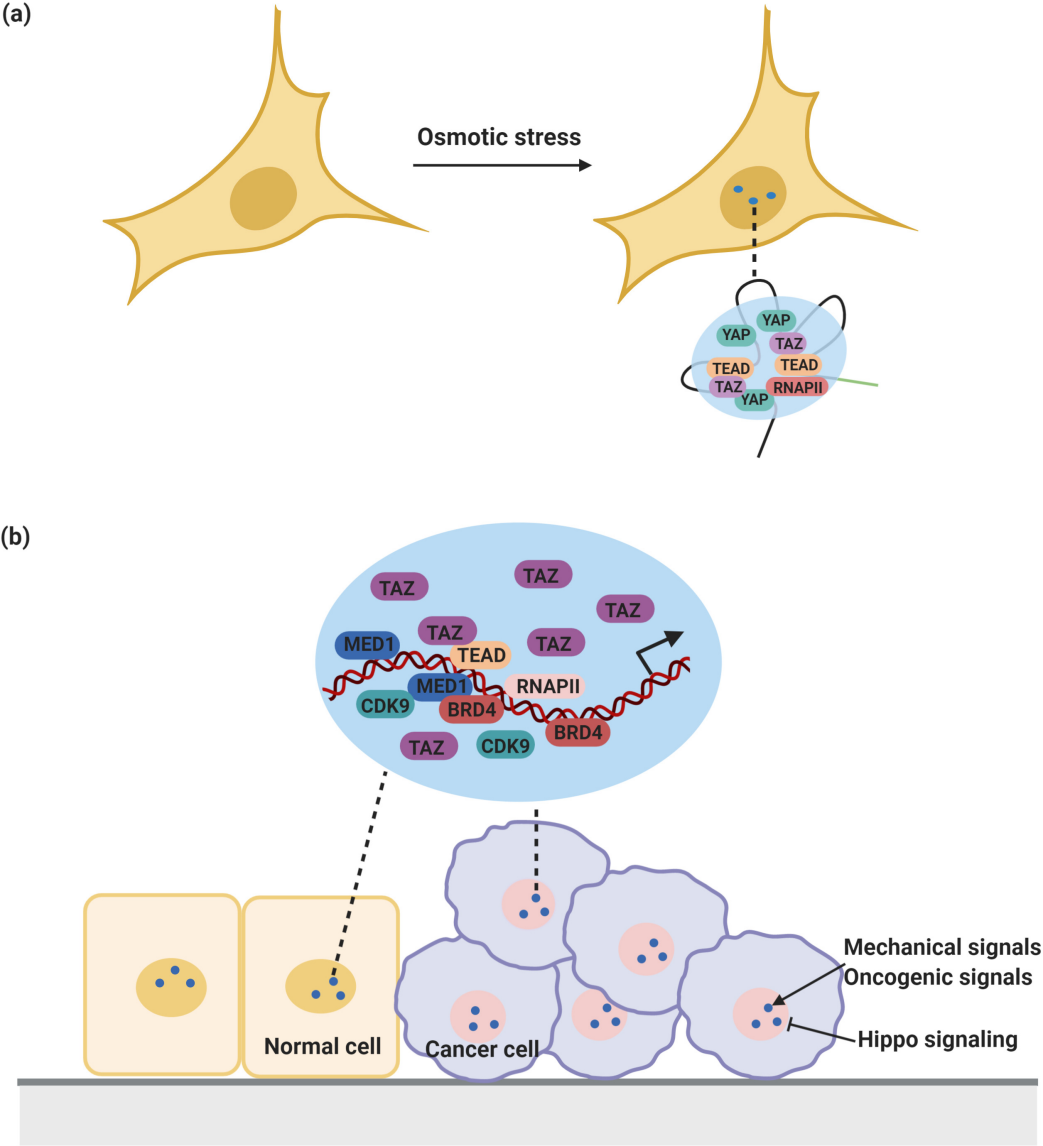
YAP/TAZ condensates in the regulation of transcription. (**a**) YAP forms nuclear condensates during osmotic stress. These YAP condensates co-localize with TAZ and TEAD and recruit RNA Pol II to trigger transcription of YAP target gene. (**b**) TAZ also forms nuclear condensates which compartmentalize TEAD4, BRD4 and MED1, RNAPII, and the transcription elongation factor CDK9 for transcription. These condensates are negatively regulated by Hippo signaling and sensitive to mechanical signals and oncogenic signals from around the environment.

Recent evidence shows that YAP and TAZ assemble into condensates in vitro and in vivo (Cai et al., 2019; Lu et al., 2020; Table 1 and Figure 4). In cells, YAP assembles into condensates at super-enhancer regions and these nuclear condensates contain TAZ but also the transcription factor TEAD1. These condensates appear to recruit RNA polymerase II to trigger the transcription of proliferative genes (Cai et al., 2019). Furthermore, YAP and TAZ mutants defective in condensate formation, displayed reduced transcriptional activity, suggesting that transcriptional activity of YAP and TAZ is associated with their ability to assemble into condensates (Cai et al., 2019; Lu et al., 2020). Importantly, TAZ-containing condensates are negatively regulated by Hippo signaling and sensitive to mechanical signals (Lu et al., 2020). In agreement with this, a growing body of evidence suggests that YAP and TAZ could function as mechanotransducers, which detect a broad range of mechanical signals and convert them into cell-specific transcriptional responses (Panciera et al., 2017). In this way, YAP and TAZ could work as signaling hubs of the tumor microenvironment. For instance, YAP and TAZ expressed in cancer cells could sense physical cues from the surrounding microenvironment and respond by driving transcriptional programs that modify the composition and physical properties of the tumor microenvironment, thus promoting tumor development (Zanconato et al., 2019). Notably, in contrast to its diffuse localization in normal breast tissue, TAZ forms nuclear condensates in breast cancer tissue (Lu et al., 2020). Therefore, it would be interesting to investigate how the mechanical properties of tumor microenvironments affect YAP or TAZ phase separation and whether YAP/TAZ-driven tumorigenesis involves condensation in cancer cells.

Condensates have not only been implicated in transcription activation, but also in transcription repression via epigenetic changes of chromatin. Indeed, epigenetic alterations in chromatin are well known to drive tumorigenesis (Flavahan et al., 2017). The first condensates that have been linked to gene silencing by heterochromatin formation are assembled from heterochromatin protein 1α (HP1α) (Larson et al., 2017; Strom et al., 2017). Another set of factors that is essential for the establishment and maintenance of facultative heterochromatin are the polycomb repressive complexes (PRC) (Tatavosian et al., 2019). These complexes also have oncogenic functions or they act as tumor suppressors, depending on the specific cancer type (Koppens and van Lohuizen, 2016). Chromobox 2 (CBX2), one subunit of Polycomb repressive complex 1 (PRC1), was recently shown to assemble into condensates that recruited the core subunits of the CBX2-PRC1 complex and directed the condensation of DNA and nucleosomes (Plys et al., 2019; Tatavosian et al., 2019; Table 1). These studies suggest that PRC1 condensates contribute to chromatin compaction, thus repressing the expression of PRC1 target genes. It will be intriguing to determine whether other epigenetic regulatory factors control the formation of gene regulatory condensates and whether impairment of epigenetic condensate control results in disease.

### Signaling condensates in the regulation of signaling transduction

Signaling pathways play an essential role in regulating gene expression. Many membrane receptors and downstream signaling molecules assemble into two-dimensional (2D) clusters upon initiation of signaling (Bienz, 2014; Wu, 2013). Well-known examples are T cell receptor signaling clusters (Ditlev et al., 2019; Huang et al., 2019; Su et al., 2016) and clusters associated with adhesion receptors (Banjade and Rosen, 2014; Beutel et al., 2019; Case et al., 2019a; Li et al., 2012). The assembly of clusters on membranes is often important for the activation of downstream signaling effectors. For instance, phase separation of phosphorylated nephrin receptor together with its downstream effector molecules NCK and N-WASP promotes actin assembly. This enhancement of actin assembly was linked to the longer membrane dwell time of N-WASP in these clusters. More generally, the dwell time of cluster components was dependent on the composition of the cluster and the concentration of the cluster constituents (Case et al., 2019b), suggesting that there is an optimal condensate composition to reach full activation. Similarly, signaling condensates formed from the phosphorylated scaffold protein linker for activation of T cells (LAT) and its two adaptors growth factor receptor-bound protein 2 (GRB2) and Son of Sevenless homolog (SOS) promoted Ras activation by increasing membrane dwell time of SOS (Huang et al., 2019). Both cases suggest that increased dwell time of signaling effectors by condensation may be a general mechanism to fully activate a signaling pathway while at the same time ensuring signaling specificity.

Notably, GRB2, SOS, and some Ras isoforms are involved in downstream signaling effectors of epidermal growth factor receptor (EGFR). EGFR is frequently mutated or overexpressed in cancer cells (Sigismund et al., 2018) and abnormal activation of Ras in the EGFR pathway results in pro-tumorigenic proliferation and migration (Martinelli et al., 2017). Given that activation of EGFR is associated with membrane-bound clusters (Liang et al., 2018), it is very likely that EGFR condensate formation regulates pro-tumorigenic activation of Ras.

Components of other oncogenic signaling pathways appear to be able to form 2D clusters at the plasma membrane (Figure 2). One example is the Wnt/β-catenin signaling pathway, which governs numerous cell fate decisions during animal development, and is deregulated in many cancers in the colon, gastric, breast, and liver (Sanchez-Vega et al., 2018; Schaefer and Peifer, 2019; Zhan et al., 2017). In the absence of a Wnt signal, β-catenin is phosphorylated by a destruction complex, which is composed of Axin, tumor suppressor adenomatous polyposis coli (APC) and some additional components. Phosphorylated β-catenin is recognized by the Cullin-based E3 Ligase SCF^βTrCP^, promoting the degradation of β-catenin by the proteasome (Stamos and Weis, 2013). In the presence of a Wnt signal, the activity of the destruction complex is repressed. Importantly, binding of Wnt to its cell surface receptors triggers the assembly of a signalosome, which is mediated by the Dishevelled (Dvl) protein (Bienz, 2020; Bilic et al., 2007; Fiedler et al., 2011; Gammons et al., 2016; Madrzak et al., 2015; Schwarz-Romond et al., 2007). The Axin complex is subsequently recruited to the signalosome, which destabilizes the destruction complex and blocks the phosphorylation of β-catenin (Stamos et al., 2014).

**Figure 5.**
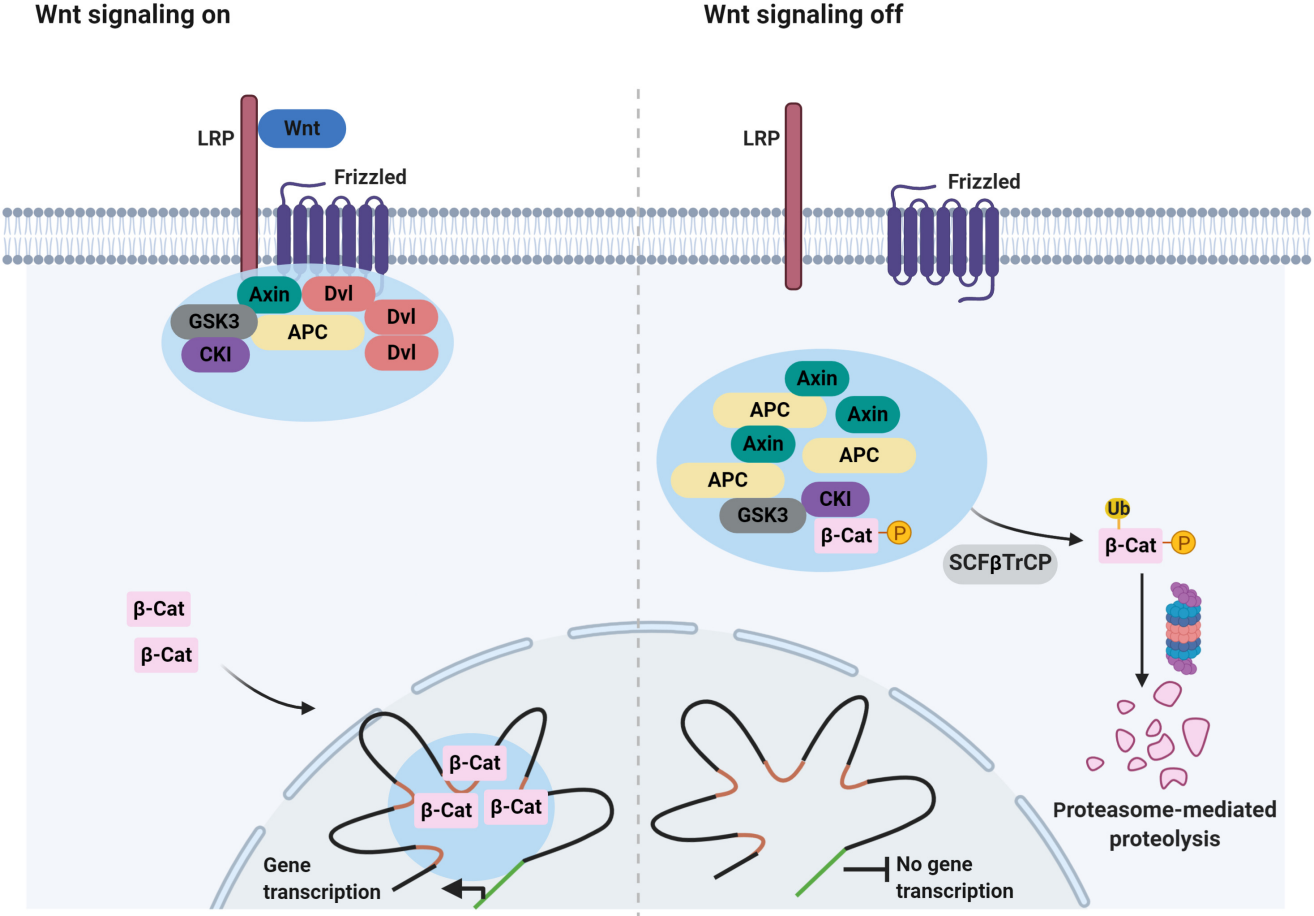
Signaling-associated condensates that may form in the Wnt/β-catenin signaling pathway. (Left) Wnt signaling triggers the assembly of 2D membrane clusters containing the receptors Frizzled and LRP as well as Dvl, Axin, and other components of destruction complex, thus disrupting destruction complex regulating degradation of β-catenin. Consequently, β-catenin accumulates and enters the nucleus, where it may localize to condensates at super-enhancers to elicit the transcription of target genes. (Right) In the absence of a Wnt ligand, Axin, and APC assemble into a destruction complex condensate that recruits kinases such as GSK3 and casein kinase I (CKI). This in turn promotes phosphorylation of β-catenin and subsequent ubiquitin-mediated degradation of β-catenin by the proteasome. Ubiquitination of phosphorylated β-catenin is mediated by the ubiquitin ligase SCFβTrCP.

Increasing evidence suggests that both the destruction complex and Wnt signalosomes have properties of condensates (Schaefer and Peifer, 2019; Figure 5). In the absence of Wnt, Axin is found in cytoplasmic puncta which also contain APC as well as other destruction complex components (Schaefer et al., 2018). By contrast, in cells receiving Wnt signals, Dvl and Axin co-localize in puncta close to the plasma membrane (Cliffe et al., 2003). The assemblies grow by fusion (Kunttas-Tatli et al., 2014; Schwarz-Romond et al., 2005) and FRAP analysis further revealed that Dvl, Axin, and APC inside the puncta exchange dynamically (Pronobis et al., 2015; Schwarz-Romond et al., 2005). Importantly, the signaling activity of Dvl is strongly correlated with the ability to form these puncta (Schwarz-Romond et al., 2005). Similarly, puncta assembly is critical for destruction complex function (Faux et al., 2008; Schaefer and Peifer, 2019). APC is required for puncta assembly and cooperates with Axin to ensure efficient β-catenin destruction (Pronobis et al., 2015). Strikingly, mutations in APC, initiate >80% of colon cancers (Zhang and Shay, 2017). The precise mechanism of how these mutations promote tumorigenesis remains to be determined. There are numbers of additional urgent questions here, for example, how APC mutations affect the assembly and properties of destruction complex condensates and the relationship between aberrant destruction complex condensates and the initiation of cancers.

Recent work has implicated condensates in another aspect of the Wnt signaling pathway. In the presence of Wnt, β-catenin accumulates in the nucleus and activates the transcription of Wnt target genes (Gammons and Bienz, 2018). Reports showed that β-catenin uses its IDRs to selectively partition into transcriptional condensates at super-enhancers (Zamudio et al., 2019; Table 1). Some cancer-related mutations in β-catenin prevent phosphorylation-dependent ubiquitination of β-catenin, leading to accumulation of β-catenin in the nucleus (Kim and Jeong, 2019). This suggests that in cancer cells, β-catenin may form aberrant nuclear condensates because of elevated protein levels and that this may promote tumorigenesis through widespread changes in gene expression.

### Protein condensates associated with protein quality control and degradation

The multi-domain adaptor protein p62/SQSTM1 (p62) is defined by its role in selective autophagy, a lysosomal degradation pathway that clears misfolded proteins and damaged organelles to maintain cellular homeostasis. The regulation of p62 is complex as p62 acts as a receptor targeting cargo for degradation but it is also itself degraded by autophagy (Sánchez-Martín et al., 2019). However, when autophagy is impaired, p62 accumulates and can activate downstream signaling pathways including mTORC1, NF-κB, and NRF2, influencing nutrient sensing, inflammation and the oxidative stress response, which may all affect tumorigenesis (Moscat et al., 2016; Sánchez-Martín et al., 2019). For instance, accumulation of p62 has been shown to accelerate the development of pancreatic cancer through activating NF-κB and NRF2 signaling (Duran et al., 2008; Ling et al., 2012; Todoric et al., 2017). Similarly, p62 accumulation in chronically damaged liver cells activates NRF2 and promotes the development of hepatocellular carcinoma (Nakagawa et al., 2014; Umemura et al., 2016).

Although the mechanism of how p62 accumulates is not fully understood, p62 is often present in cellular inclusion bodies. Inclusion bodies in the brain include Lewy bodies, neurofibrillary tangles, and huntingtin aggregates; inclusion bodies in the liver include Mallory-Denk bodies, intracytoplasmic hyaline bodies, and α1 antitrypsin aggregates (Komatsu et al., 2007; Yamamoto and Simonsen, 2011). Intriguingly, recent studies have shown that p62 assembles together with ubiquitinated proteins into condensates (Table 1), and the formed condensates are subsequently engulfed by autophagosomes and degraded (Sun et al., 2018; Zaffagnini et al., 2018). Another study found that p62 assembles into condensates together with mutant KEAP1 proteins and the transcription factor NRF2, thereby affecting NRF2-driven transcription (Cloer et al., 2018). Although this remains to be determined, it is tempting to speculate that p62 condensates are involved in the formation and autophagy-mediated disposal of various cellular condensates that promote or inhibit tumorigenesis.

### Dysregulation of membraneless compartments in cancer

**Figure 6.**
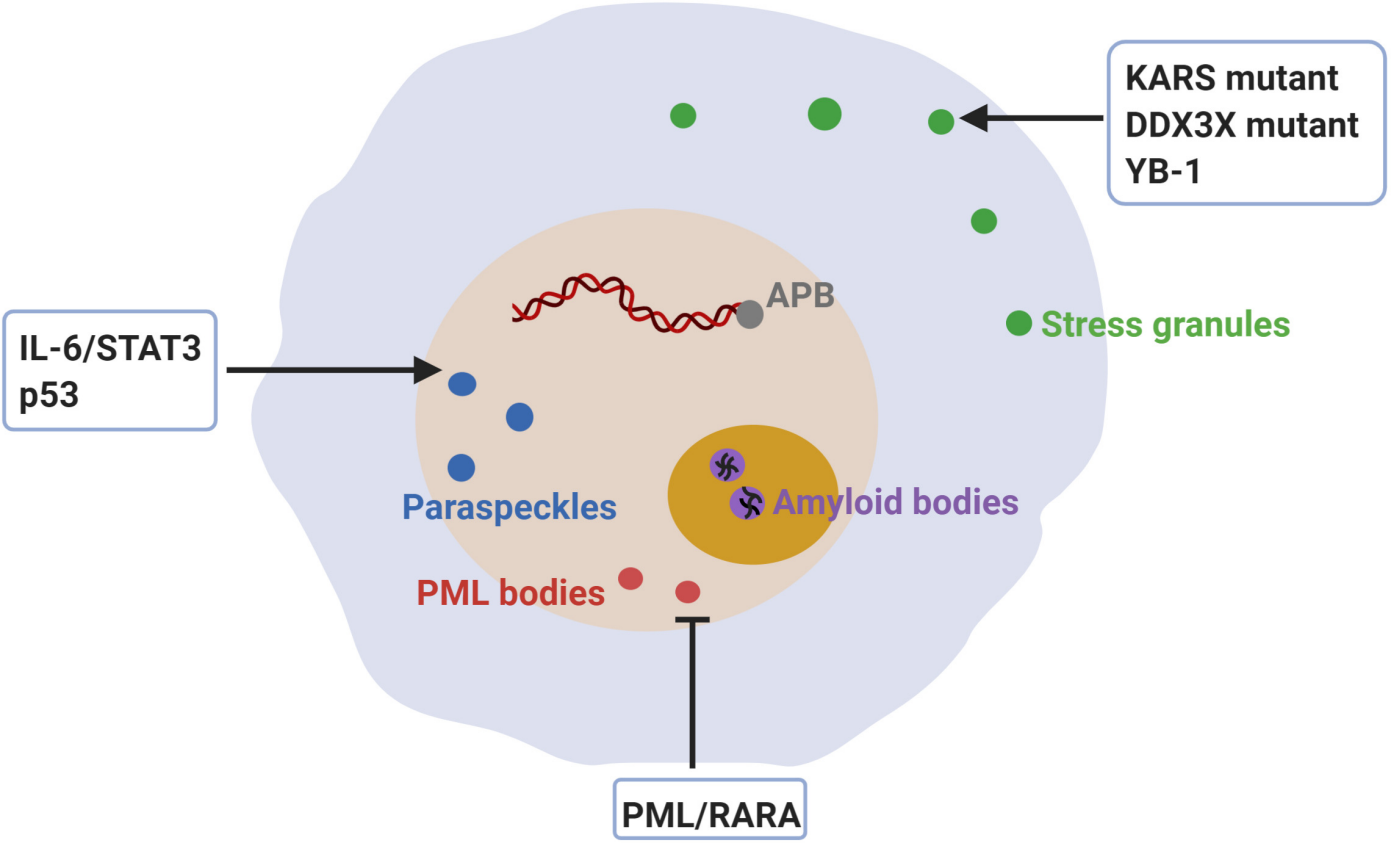
Dysregulation of membraneless compartments in cancer. Aberrant assembly of SGs, PML bodies or paraspeckles may arise from dysregulation of or mutations in cancer-related proteins, thus promoting tumorigenesis. For instance, KRAS mutations promote SG hyper-assembly and this has been shown to confer a fitness advantage to cancer cells (Grabocka and Bar-Sagi, 2016). Likewise, mutations in DDX3X cause SG hyper-assembly and this has been shown to impair protein synthesis in medulloblastomas (Valentin-Vega et al., 2016). Moreover, YB-1 promotes SG assembly, which has been linked to increased invasion and metastasis (Somasekharan et al., 2015). The expression of PML/RARA leads to disruption of PML body assembly and deregulated transcriptional control of senescence and differentiation in acute promyelocytic leukemia (APL) (de Thé and Chen, 2010; Dos Santos et al., 2013). Furthermore, disruption of PML bodies contributes to APL pathogenesis by increasing genome instability (Voisset et al., 2018). Recombination-based alternative lengthening of telomeres (ALT) is a key mechanism for telomerase-negative cancer cells to maintain the telomere stability and the capability for unlimited proliferation (Bryan et al., 1997). ALT-associated PML bodies (APB) facilitate telomere maintenance and thus promote cancer cell immortality (Zhang et al., 2020). IL-6/STAT3 signaling promotes paraspeckles formation, which favors overactivation of STAT3 in human hepatocellular carcinoma (HCC) (Wang et al., 2018c). Paraspeckle assembly induced by p53 has been shown to inhibit cancer initiation in pancreatic cancer models (Mello et al., 2017). Finally, inhibition of amyloid body assembly has been shown to promote tumor tissue growth (Audas et al., 2016).

Accumulating evidence suggests that aberrant assembly of condensates is associated with cancer. How aberrant assembly and dysregulation of well-known membraneless compartments that form through condensation may promote tumorigenesis will be discussed in this section (Figure 6).

### Stress granules

SGs, a type of stress-induced membraneless compartment, promote cell survival during stress conditions and have been shown to be formed by phase separation (Guillén-Boixet et al., 2020; Molliex et al., 2015; Patel et al., 2015; Sanders et al., 2020; Yang et al., 2020). Due to the high metabolic demands of proliferation, cancer cells usually exist in a unique microenvironment characterized by hypoxia, high levels of reactive oxygen species, and nutrient starvation (Ackerman and Simon, 2014), conditions which activate the cellular stress response and trigger SG assembly. The assembly of SG promotes cancer cell adaption to adverse microenvironments and enhances cancer cell resistance to apoptosis by accumulating anti-apoptosis molecules (Arimoto et al., 2008; Thedieck et al., 2013).

Increased assembly of SG is observed in different kinds of cancers and modulated by cancer-related proteins (Figure 2). For instance, KRAS is a member of the RAS oncogene family, the most frequently mutated oncogene family in human cancers (Cox et al., 2014), has been linked to SG assembly. KRAS mutations are detected in many highly malignant cancers, such as pancreatic ductal adenocarcinoma, colorectal adenocarcinoma, and lung adenocarcinoma (Cox et al., 2014). SG assembly was found to be induced in transformed or cancerous cells expressing mutant *Kras*, and this was shown to depend on the secretion of 15-deoxy-delta 12, 14 prostaglandin J2 (15-d-PGJ2), a lipid that inactivates the eukaryotic initiation factor eIF4A (Kim et al., 2007; Grabocka and Bar-Sagi, 2016; Table 1 and Figure 6). Importantly, inhibiting SG assembly and prostaglandin synthesis caused mutant *Kras* cells to be more sensitive to stress. Thus, mutant *Kras* establishes a more stress-resistant cellular condition through SG assembly and this confers a fitness advantage to cancer cells and presumably also resistance to chemotherapeutics.

Another class of proteins linked to cancer are confirmed regulators of SG assembly. For instance, members of RNA-dependent DEAD-box helicases (DDXs) are conserved regulators of RNA-containing condensates like SGs (Hondele et al., 2019; Tauber et al., 2020). The ATPase activity of DDXs is required for controlling the partitioning of RNA between different types of condensates. One example is DDX3X, which is mutated in many human tumor types (Valentin-Vega et al., 2016). In medulloblastomas (MB), mutations in DDX3X affect RNA-stimulated ATP hydrolysis and this causes SG hyper-assembly even under non-stress conditions, which results in impairing global translation (Valentin-Vega et al., 2016; Table 1 and Figure 6). In agreement with this, deleting the N-terminal IDR of MB-associated DDX3X prevented SG hyper-assembly and reversed the translation inhibitory effect (Valentin-Vega et al., 2016). In agreement with this, accumulating evidence indicates that deregulation of translation promotes cellular transformation and tumor development (Ruggero, 2013). Therefore, perturbations in translational control caused by aberrant SG assembly may be another pathway that promotes tumorigenesis.

Another SG component that has been shown to facilitate SG assembly is Y-box binding protein 1 (YB-1). YB-1 is a member of a highly conserved cold shock domain (CSD) family and implicated in a wide variety of cellular function, such as translational regulation, DNA repair, and stress responses (Kohno et al., 2003). Increased protein levels of YB-1 are highly correlated with cancer progression and poor prognosis (Lasham et al., 2013). YB-1 was recently shown to promote SG assembly by translationally upregulating G3BP1 which is essential for SG assembly (Guillén-Boixet et al., 2020; Sanders et al., 2020; Somasekharan et al., 2015; Yang et al., 2020; Table 1 and Figure 6). Knockdown of G3BP1 severely impairs SG assembly and inhibits invasion and metastasis (Somasekharan et al., 2015). Thus, the role of YB-1 in cancer progression may be linked to SG assembly. However, how SG assembly promotes invasion and metastasis remains unclear and this aspect should be investigated by building on our improved understanding of condensate assembly.

### PML bodies

PML bodies are stress-sensitive nuclear condensates (Banani et al., 2016; Zhu and Brangwynne, 2015). They are involved in transcriptional regulation, protein modification, apoptosis, cellular senescence, cell cycle progression, angiogenesis, and protein quality control (Hsu and Kao, 2018; Mediani et al., 2019). The formation of the PML body is driven by PML:PML interactions (Huang et al., 2014) and SUMOylation of PML, which promotes the recruitment of proteins containing SUMO-interacting motifs (SIMs) to PML bodies (Banani et al., 2016). In addition to SUMOylation-related enzymes (UBC9, RNF4), PML bodies also contain many other enzymes, such as HIPK2 kinase, the CBP or MOZ acetyl transferases (Lallemand-Breitenbach and de Thé, 2018). Importantly, PML body formation regulates PTMs on p53, which are required for full p53 activity and oncogene-induced senescence (Ferbeyre et al., 2000; Pearson et al., 2000).

Dysregulation of PML bodies is associated with diverse cancers (Hsu and Kao, 2018; Figure 2). In acute promyelocytic leukemia (APL), PML is fused with full-length Retinoic Acid Receptor-alpha (RARA) because of a chromosomal translocation (de Thé et al., 1990). The expression of PML/RARA leads to disruption of PML bodies and deregulation of transcriptional programs that control senescence and differentiation (de Thé and Chen, 2010; Dos Santos et al., 2013; Table 1 and Figure 6). Therapeutic approaches have been developed to treat APL through a combination of retinoic acid (RA) and arsenic trioxide therapies. RA and arsenic trioxide induce PML body formation and this in turn promotes p53-driven senescence, which is required for APL eradication (Ablain et al., 2013; Ablain et al., 2014; de Thé et al., 2017). Thus, defective PML body formation promotes APL progression at least in part because APL cells cannot activate p53-driven senescence. Moreover, recent work reveals that aberrant PML body formation contributes to APL pathogenesis by increasing genome instability (Voisset et al., 2018). PML body disruption was shown to cause chromosome abnormalities and impair DNA damage response pathways.

Telomere maintenance is critical for a cancer cell to achieve the ability to proliferate in an unlimited manner (Blasco, 2005). Telomerase-negative cancer cells employ a mechanism known as recombination-based alternative lengthening of telomeres (ALT) to maintain telomere length and stability (Bryan et al., 1997). In ALT cancer cells, PML bodies associate with telomeres, their protective sheltering proteins TRF1/2 and several DNA repair proteins to form ALT-associated PML bodies (APB) (Osterwald et al., 2015; Yeager et al., 1999). A recent study suggests that the formation of APBs is driven by phase separation, thus promoting the clustering of telomere repeats and telomere lengthening (Zhang et al., 2020; Figure 6). Consequently, knocking down the PML body component PML inhibited APB formation and caused telomere shortening (Draskovic et al., 2009; Loe et al., 2020; Osterwald et al., 2015). Together, this suggests that APBs facilitate ALT telomere maintenance, eventually allowing cancer cells to grow indefinitely and become immortal.

### Paraspeckles

Paraspeckles are nuclear bodies which regulate gene expression (Fox et al., 2018). The ncRNA scaffold NEAT1_2 drives the assembly of paraspeckles by interacting with essential paraspeckle proteins, such as NONO, SFP, FUS and RBM14 (Yamazaki et al., 2018; Table 1). Importantly, abnormal assembly of paraspeckles has been described in diverse cancers (Adriaens et al., 2016; Figure 2). In human hepatocellular carcinoma (HCC), inflammation-related IL-6 signaling increases paraspeckle formation by promoting the transcription of NEAT1_2, which is medicated by the transcription factor STAT3 and H3K4me3 histone modifications (Wang et al., 2018c; Figure 6). Increased paraspeckle formation promotes further STAT3 activation via sequestering negative regulators of STAT3 and tumor repressors, thus causing a vicious cycle that drives further paraspeckle assembly. Importantly, over-activation of STAT3 induces the transcription of various genes involved in cellular survival, inflammation, epithelial to mesenchymal transition, and cancer stem cell maintenance (Yu et al., 2014), all of which promote tumor progression.

Beyond the oncogenic role of paraspeckles in cancer progression, other studies have suggested that paraspeckles acts as a tumor suppressor in certain contexts. The non-coding, paraspeckle-associated RNA NEAT1 is induced by p53 in response to various p53-activating signals (Mello et al., 2017; Figure 6). Additionally, NEAT1 overexpression suppresses the transformation pancreatic cancer cells, and this effect was associated with an increase in the number of paraspeckles. Conversely, NEAT1 deficiency was shown to impair paraspeckle formation and promote pancreatic cancer initiation. Although this remains to be investigated, paraspeckles could regulate transcription factors associated with specific gene expression programs, thus promoting the expression of tumor suppressors and increasing the expression of developmental pancreas genes.

In addition to STAT3 and p53, NEAT1 has been shown to be regulated by other cancer-related transcription factors, such as HIF-2α (Choudhry et al., 2015), Oct4 (Jen et al., 2017), PML/RARA (Zeng et al., 2014), and c-Myc (Zeng et al., 2018). The example of PML/RARA is particularly interesting, because it not only represses NEAT1 expression (Zeng et al., 2014), but also disrupts PML bodies (discussed above). This suggests that the oncoprotein PML/RARA promotes aberrant assembly of two nuclear condensates, PML bodies and paraspeckles, to drive tumorigenesis.

Finally, NEAT1 has been reported to regulate many cancer-related microRNAs whose targets mRNAs are involved in cell proliferation, migration, invasion, metastasis, EMT, stem cell-like phenotype, chemoresistance and radioresistance (Dong et al., 2018). For instance, NEAT1 promotes metastasis by abolishing microRNA-382–3 p-mediated suppression of Rho Associated Coiled-Coil Containing Protein Kinase 1 (ROCK1) (Liu et al., 2018). However, it remains to elucidate how aberrant assembly of paraspeckles affects the activity of microRNAs.

### Amyloid bodies

Amyloid bodies are stress-induced storage compartments in the nucleolus. The formation of amyloid bodies is seeded by non-coding RNA transcribed from loci of the rDNA intergenic spacer (rIGSRNA) and likely driven by complex coacervation of low-complexity rIGSRNA and short cationic domains in amyloid converting motif (ACM) (Wang et al., 2018b). Acidosis stress as found in tumor microenvironments was shown to induce the assembly of amyloid bodies, which involved the recruitment of many proteins involved in cell cycle regulation and DNA synthesis to amyloid bodies (Audas et al., 2016). It was postulated that amyloid body formation induces a protective state of cellular dormancy that may help cancer cells to adapt to the harsh tumor microenvironment (Wang et al., 2019). In agreement with this, inhibition of amyloid body formation by knockdown of rIGS28RNA prevented tumor dormancy and led to larger tumor sizes in cancer mouse models (Audas et al., 2016; Figure 6). However, how amyloid bodies promote tumorigenesis remains to be shown (Figure 2).

### Conclusion and future perspectives

Condensates have now been implicated in almost all fundamental processes in living cells. Given the importance of condensates for cellular physiology and our increasingly better understanding of condensate assembly and function, we expect that condensate research will make an important contribution to unraveling the complex biology of cancers in the coming years. One example of the increasingly important role of condensates is the demonstration that transcription is regulated by condensates. Numerous cancer-related proteins function as transcription factors and assembly of these transcription factors into aberrant condensates could drive various hallmarks of cancer cells, such as their ability to proliferate.

However, cancer cells acquire various other capabilities during tumorigenesis, such as induction of angiogenesis, activation of invasion and metastasis, deregulation of cellular energetics, avoidance of immune detection, and destabilization of the genome (Hanahan and Weinberg, 2011). Increasing evidence implicates condensates also in these processes, with confirmed roles for example in mitosis (Jiang et al., 2015; Rai et al., 2018), immune cell signal transduction (Su et al., 2016), chromatin organization (Gibson et al., 2019), and cell adhesion (Beutel et al., 2019). A future challenge will be to determine whether the corresponding physiological condensates are misregulated in cancer cells. For example, does the spindle pole regulator BuGZ (Jiang et al., 2015) form condensates in cancer cells and does aberrant BuGZ assembly promote hyperproliferation or metastasis? Does aberrant assembly of zona occludens condensates (Beutel et al., 2019) contribute to cancer cell invasion and metastasis? Such questions underscore the importance of investigating cancer-associated processes though the lens of condensate biology. Furthermore, illuminating how specific cancer-associated mutations promote aberrant phase behavior of proteins and promote condensate dysfunction will yield entirely new molecular mechanisms underlying cancer initiation and progression. We expect that the growing field of condensate biology will not only create more knowledge about the molecular underpinnings of cancer but it will also accelerate the development of new therapies, thus bringing us closer to the goal of curing cancer.
